# New Insights in Addressing Cerebral Small Vessel Disease: Association With the Deep Medullary Veins

**DOI:** 10.3389/fnagi.2020.597799

**Published:** 2020-12-01

**Authors:** Zhihua Xu, Fangfei Li, Bing Wang, Dengxiang Xing, Yusong Pei, Benqiang Yang, Yang Duan

**Affiliations:** ^1^Department of Radiology, TongDe Hospital of Zhejiang Province, Hangzhou, China; ^2^Department of Radiology, Center for Neuroimaging, General Hospital of Northern Theater Command, Shenyang, China; ^3^General Hospital of Northern Theater Command Training Base for Graduate, Dalian Medical University, Shenyang, China; ^4^Department of Scientific Research, General Hospital of Northern Theater Command, Shenyang, China; ^5^Center for Medical Data, General Hospital of Northern Theater Command, Shenyang, China; ^6^General Hospital of Northern Theater Command Training Base for Graduate, Jinzhou Medical University, Shenyang, China; ^7^Department of Radiology, The General Hospital of Northern Theater Command, Shenyang, China; ^8^General Hospital of Northern Theater Command Training Base for Graduate, China Medical University, Shenyang, China

**Keywords:** deep medullary vein, cerebral small vessel disease, white matter hyperintensity, cerebral microbleed, perivascular space, lacunar infarct

## Abstract

**Objective:**

To assess the suitability of deep medullary vein visibility in susceptibility weighted imaging—magnetic resonance imaging studies as a method for the diagnosis and evaluation of cerebral small vessel disease progression.

**Methods:**

A total of 92 patients with CSVD were enrolled and baseline clinical and imaging data were reviewed retrospectively. Neuroimaging biomarkers of CSVD including high-grade white matter hyperintensity (HWMH), cerebral microbleed (CMB), enlarged perivascular space (PVS), and lacunar infarct (LI) were identified and CSVD burden was calculated. Cases were grouped accordingly as mild, moderate, or severe. The DMV was divided into six segments according to the regional anatomy. The total DMV score (0–18) was calculated as the sum of the six individual segmental scores, which ranged from 0 to 3, for a semi-quantitative assessment of the DMV based on segmental continuity and visibility.

**Results:**

The DMV score was independently associated with the presence of HWMH, PVS, and LI (*P* < 0.05), but not with presence and absence of CMB (*P* > 0.05). Correlation between the DMV score and the CSVD burden was significant (*P* < 0.05) [OR 95% C.I., 1.227 (1.096–1.388)].

**Conclusion:**

The DMV score was associated with the presence and severity of CSVD.

## Introduction

Cerebral small vessel disease (CSVD) is a generic term that refers to the collection of pathological, clinical, and neuroimaging changes affecting the small vessels of the brain (Pantoni, 2010). CSVD is common in the population, especially in the elderly, and is implicated in up to a quarter of all strokes (Suzuyama and Yakushiji, 2020). Additionally, CSVD is widely regarded as a common cause of cognitive decline and dementia (Peng, 2019).

Magnetic resonance imaging (MRI) is a useful approach to assessment of CSVD. The imaging features of CSVD (Wardlaw et al., 2013) include white matter hyperintensity (WMH), cerebral microbleed (CMB), enlarged perivascular space (PVS), and lacunar infarct (LI). A CSVD score integrating these four radiologic features can suggest the severity of CSVD, and may even predict the risk of cognitive decline, dementia, and subsequent stroke (Amin Al Olama et al., 2020; Suzuyama and Yakushiji, 2020).

However, the precise underlying pathophysiological mechanism of CSVD remains unclear. Damage to small arteries has been identified as a common pathological change in CSVD and may play a key role in the onset of CSVD (Pantoni, 2010; Wardlaw et al., 2013). Earlier studies (Dolui et al., 2019; Yu et al., 2020) were mainly focused on cerebral blood flow and small arteries, while a small body of research (Fulop et al., 2019) has considered the importance of the cerebral veins in balancing and stabilizing cerebral blood flow, and has demonstrated that alterations of the cerebral veins may also trigger or aggravate CSVD. Even small cerebral veins can be the site of an initial inflammatory response, leading to changes in the content of interstitial fluid in the brain and blood vessels and damage to the brain parenchyma (Chung and Hu, 2010). Thus, further study to explore the association between cerebral veins and CSVD is necessary.

Susceptibility weighted imaging (SWI) is a unique MRI technique that shows cerebral veins well *in vivo*. Because there are few developmental variations in the deep medullary vein (DMV), the DMV has been widely studied in ischemic stroke (Mucke et al., 2015; Han et al., 2016; Duan et al., 2018; Xu et al., 2019). CSVD is a disease affected small vessels of the brain resulting in changes of microenvironment, such as blood oxygen saturation, and leading to DMV changes. Thus, we hypothesized that the presence and burden of CSVD may be associated with DMV changes. However, the neuroimaging biomarkers of CSVD (WMH, CMB, PVS, and LI) and total burden of CSVD correlated with DMV changes did not observed thoroughly. So that, we aim to assess the suitability of deep medullary vein visibility in SWI—MRI studies as a method for the diagnosis and evaluation of CSVD progression in this study.

## Materials and Methods

### Patients

**FIGURE 1:**
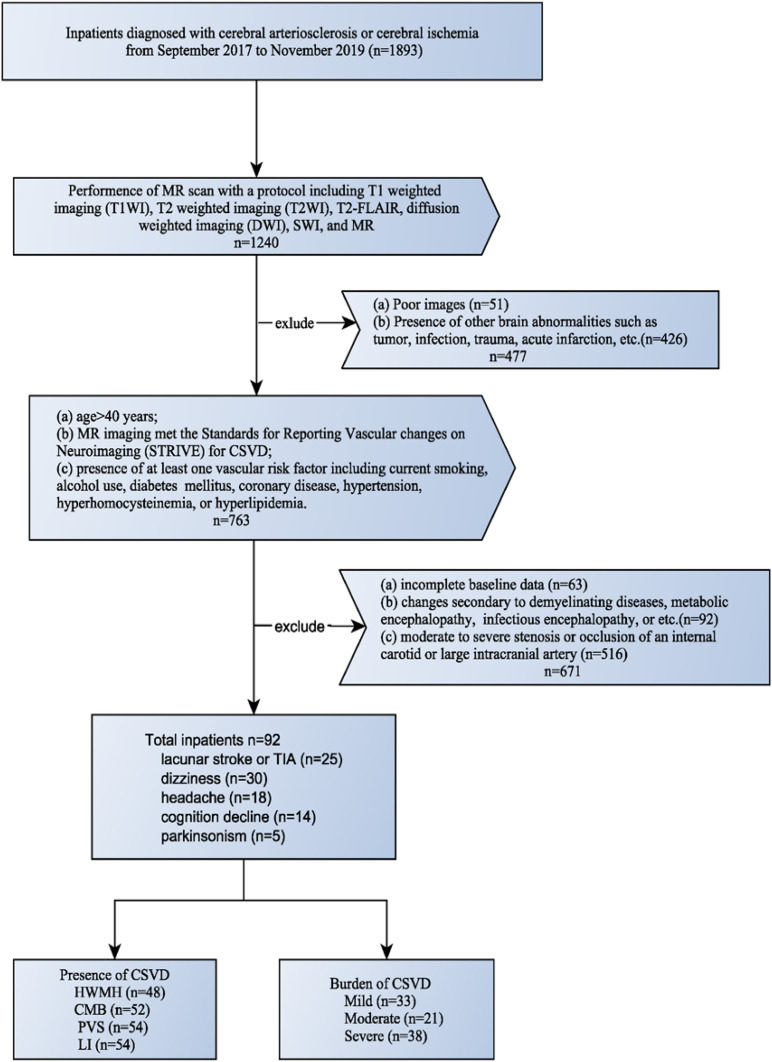
Flowchart of enrollment of study patients.

The protocol for this study was approved by the Institutional Review Board of General hospital of Northern Theater Command. All patients or their legally authorized representatives provided written informed consent prior to participation in this study. The clinical and imaging data of patients with CSVD were collected and reviewed from September 2017 to November 2019. All enrolled patients had MRIs performed for various neurological indications (Figure 1) and those with presence of cerebral arteriosclerosis or cerebral ischemia were included. The inclusion criteria were: (a) age > 40 years; (b) MR protocol including T1 weighted imaging (T1WI), T2 weighted imaging (T2WI), T2-FLAIR, diffusion-weighted imaging (DWI), SWI, and MRA; (c) MR imaging met the Standards for Reporting Vascular changes on Neuroimaging (STRIVE) for CSVD(Wardlaw et al., 2013); (d) presence of at least one vascular risk factor including current smoking, alcohol use, diabetes mellitus, coronary disease, hypertension, hyperhomocysteinemia, or hyperlipidemia. The exclusion criteria were: (a) incomplete baseline data; (b) changes secondary to demyelinating diseases, metabolic encephalopathy, infectious encephalopathy, or etc.; (c) presence of other brain abnormalities such as tumor, infection, trauma, acute infarction, etc.; (d) moderate to severe stenosis or occlusion of an internal carotid or large intracranial artery; (e) patients with hereditary CSVD. The flowchart of enrollment of study patients was shown in Figure 1.

### Clinical Information

The sex, age, risk factors for CSVD including current smoking, alcohol use, diabetes mellitus, coronary disease, hypertension, blood pressure (systolic, diastolic, pulse pressure, mmHG), hyperhomocystinemia, hyperlipidemia of all patients were calculated on admission.

### Magnetic Resonance Imaging Protocol

All patients underwent multimodal MRI on a 3.0T Discovery MR750 scanner (General Electric Healthcare, Chicago, IL, United States) using an eight-channel phased-array head coil. The relevant important parameters were SWI: repetition time = 27 ms; echo time = 20 ms; flip angle = 10°; slice thickness = 2 mm; intersection gap = 0 mm; field of view = 24 × 24 cm^2^; and matrix number = 512 × 512.

### Presence and Severity of Cerebral Small Vessel Disease

All images were reviewed separately by two neuroradiologists. Disagreements were resolved by consensus. We identified WMH, CMB, PVS, and LI according to STRIVE (Wardlaw et al., 2013) and estimated the CSVD burden. WMH was defined as abnormal hyperintensity of periventricular white matter or deep white matter on T2 FLAIR images. The extent of WMH was assessed and scored by using the Fazekas scoring system, and high-grade white matter hyperintensities (HWMH) were defined by Fazekas score of ≥ 2 in the periventricular white matter and/or ≥ 2 in deep white matter. CMBs were defined as homogeneous hypointensities with an average diameter of 3–5 mm on SWI after excluding calcification, vascular cross section, and abnormal iron deposits. CMBs were counted and graded as grade 0 = no CMB; grade 1 = 1–2 CMBs; and grade 2 = more than 2 CMBs. PVS enlargement was defined by small dot-like or linear fluid signals accompanied by small blood vessels on MR images. High grade PVS (HPVS) meant that the number of enlarged PVSs at the level of the maximum number of PVSs in the unilateral vasal ganglia was more than 10. LI were defined as round or ovoid subcortical lesions measuring 3–15 mm in diameter that manifested as hyperintense lesions on T2WI and as hypointense lesions on T1WI.

The CSVD burden was assessed as total CSVD score, which is based on an ordinal scale ranging from 0 to 4 depending on the absence of presence (0 or 1) of each of the four CSVD features (HWMH, CMB, HPVS, and LI), and patients were divided into three groups, mild (total CSVD score = 0 or 1), moderate (total CSVD score = 2), and severe (total CSVD score = 3 or 4), according to the CSVD burden. For example, if one was detected with multiple CMBs and HWMH (without HPVS, and LI), the total score of CSVD is of 2. Thus, the burden of CSVD is classified into moderate group.

### Deep Medullary Veins Score

**FIGURE 2:**
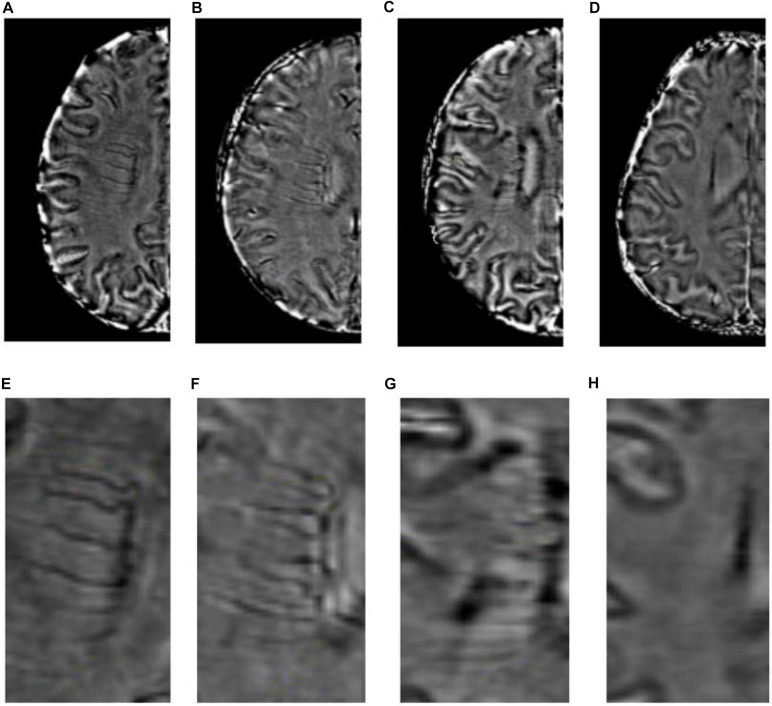
Deep medullary veins scoring system. **(A,E)** A score of 0 is assigned for each continuous and prominently visible vein. **(B,F)** A score of 1 indicates that the vein is continuous and with unequivocal visibility, but at least one vein has inhomogeneous signal. **(C,G)** A score of 2 indicates that at least one vein is not continuous and with faint visibility, presenting with spot-like hypointensity. **(D,H)** A score of 3 is assigned if the DMV is not visible.

DMV scores were assessed on the SWI sequences. We assessed DMVs on five consecutive periventricular slices (10 mm thick) of SWI phase images from the level of the ventricles immediately above the basal ganglia to the level of the ventricles immediately disappeared for each patient, considering that these slices cover most of the DMVs. According to the regional anatomy (Zhang et al., 2017), the DMV is divided into six segments, frontal, parietal, and occipital (bilateral, respectively). Each segment is scored separately with a DMV score of 0–3 for semi-quantitative assessment of the DMV based on observing the continuity and visibility (Zhang et al., 2017). The DMV score is 0 when there are no interruptions and the segment is continuous and prominently visible. A score of 1 indicates a continuous vein with unequivocal visibility, but with an inhomogeneous signal in at least one vein. A score of 2 indicates that at least one vein is not continuous and has faint visibility, presenting with spot-like hypointensity. A score of 3 is assigned when the DMV is invisible (Figure 2). The total DMV score ranges from 0 to 18 and is the sum of the DMV scores of each of the six segments. A score of 0 indicates a prominent DMV, while a score of 18 refers to an inapparent DMV. All images were reviewed separately by two neuroradiologists who were completely blinded to the subjects’ clinical data and disease state. Disagreements were resolved by consensus.

### Statistical Analysis

Categorical variables are shown as frequencies and percentages. Continuous, normally distributed data are described as means and standard deviations (SD), and non-parametric data are described as medians and interquartile ranges (IQR). Differences in normally distributed data were analyzed using *t*-test; differences in categorical variables were analyzed using Chi-square test; and differences in non-parametric data were analyzed using Mann Whitney U test or Kruskal-Wallis test. First, the univariate analysis was performed to compared the baseline clinical and imaging characteristics between presence and absence of HWMH, CMB, PVS, and LI, as well as among burden of CSVD. Then, binary logistic regression analysis was performed to identify independent factors associated with the presence of CSVD (HWMH, CMB, PVS, and LI). The correlations were adjusted with related factors according to univariate analysis. Finally, ordinal logistic regression analysis was performed to identify independent factors associated with the CSVD burden. Statistical significance was defined as *P* < 0.05. The data were analyzed using the Statistical Package for Social Sciences for Windows, Version 20 (IBM Corp., Armonk, NY, United States).

## Results

There were 92 patients with CSVD enrolled in this study. Their mean age was 65 ± 11 years old. Among them, HWMH were seen in 48 (52.17%), CMB in 52 (56.52%), and HPVS and LI in 54 (58.69%). The median (IQR) CSVD and DMV scores were 2 (1, 3) and 7.00 (5.00, 10.25), respectively. The agreement between the two neuroradiologists was excellent for total DMV score (the sum of the DMV scores of each of the six segments) on SWI (κ = 0.837) and for burden of CSVD on T2-FLAIR images (κ = 0.918).

### Presence of Cerebral Small Vessel Disease

#### Deep Medullary Veins Score and HWMH

The clinical and imaging characteristics and comparisons among study patients according to the presence of CSVD are shown in Table 1. After univariate analysis, age, CMB grade, PVS, LI, and DMV score significantly differed between presence (*n* = 48) and absence (*n* = 44) of HWMH (*P* < 0.05); however, it did not differ in sex, current smoking, alcohol use, diabetes mellitus, coronary disease, hypertension, blood pressure (systolic, diastolic and pulse pressure), hyperhomocystinemia, hyperlipidemia between presence and absence of HWMH (*P* > 0.05). After binary logistic regression, age, CMB grade, and DMV score were independently associated with HWMH (Table 2).

**TABLE 1:** Clinical and imaging characteristics and comparisons among study patients according to the presence of cerebral small vessel disease.

	**HWMH**		***P*-value**	**CMB**		***P*-value**	**PVS**		***P*-value**	**LI**		***P*-value**
	**No (*n* = 44)**	**Yes (*n* = 48)**		**No (*n* = 40)**	**Yes (*n* = 52)**		**No (*n* = 38)**	**Yes (*n* = 54)**		**No (*n* = 38)**	**Yes (*n* = 54)**	
Age, years	62 ± 11	68 ± 10	0.003	66 ± 11	65 ± 11	0.798	58 ± 10	70 ± 9	<0.001	58 ± 10	70 ± 9	<0.001
Sex, M	29 (65.9)	35 (72.9)	0.615	26 (65.0)	38 (73.1)	0.544	25 (65.8)	39 (72.2)	0.667	25 (65.8)	39 (72.2)	0.667
**CSVD**												
CMB	20 (45.5)	32 (66.7)	0.066				17 (44.7)	35 (64.8)	0.089	17 (44.7)	35 (64.8)	0.089
Grade of CMB	0 (0, 1)	1 (0, 3)	0.003				0 (0, 1)	1 (0, 2)	0.063	0 (0, 1)	1 (0, 2)	0.063
WMH				1 (1, 3)	3 (1, 4)	0.013	1 (0, 3)	3 (1, 4)	<0.001	1 (0, 3)	3 (1, 4)	<0.001
Periventricular WMH				1 (1, 2)	1 (1, 2)	0.152	1 (0, 1)	2 (1, 2)	0.001	1 (0, 1)	2 (1, 2)	0.001
Deep WMH				0 (0, 1)	2 (0, 2)	0.002	0 (0, 1)	1 (0, 2)	0.003	0 (0, 1)	1 (0, 2)	0.003
Lacunar infarction	17 (38.6)	37 (77.1)	< 0.001	15 (37.5)	39 (75.0)	0.001	14 (36.8)	40 (74.1)	0.001			
PVS	18 (40.9)	36 (75.0)	0.002	19 (47.5)	35 (67.3)	0.089				0 (0.0)	54 (100.0)	< 0.001
**Risk factors for CSVD**												
Current smoking	17 (38.6)	21 (43.8)	0.775	13 (32.5)	25 (48.1)	0.197	16 (42.1)	22 (40.7)	1.000	16 (42.1)	22 (40.7)	1.000
Alcohol use	15 (34.1)	18 (37.5)	0.902	9 (22.5)	24 (46.2)	0.034	11 (28.9)	22 (40.7)	0.347	11 (28.9)	22 (40.7)	0.347
Diabetes mellitus	22 (50.0)	27 (56.2)	0.696	20 (50.0)	29 (55.8)	0.735	18 (47.4)	31 (57.4)	0.46	18 (47.4)	31 (57.4)	0.46
Coronary disease	10 (22.7)	11 (22.9)	1.000	8 (20.0)	13 (25.0)	0.752	9 (23.7)	12 (22.2)	1.000	9 (23.7)	12 (22.2)	1.000
Hypertension	35 (79.5)	43 (89.4)	0.547	31 (77.5)	47 (93.4)	0.162	31 (81.6)	47 (87.0)	0.725	31 (81.6)	47 (87.0)	0.725
**Blood pressure, mmHG**												
Systolic	148 ± 18	149 ± 22	0.925	140 ± 19	156 ± 18	<0.001	146 ± 22	150 ± 19	0.449	144 ± 18	152 ± 21	0.449
Diastolic	85 ± 10	88 ± 13	0.219	81 ± 10	91 ± 12	< 0.001	88 ± 13	86 ± 11	0.625	85 ± 11	89 ± 12	0.625
Pulse pressure	63 ± 15	60 ± 17	0.533	58 ± 15	64 ± 17	0.071	58 ± 17	64 ± 15	0.091	60 ± 16	62 ± 16	0.475
Hyperhomocystinemia	6 (13.63)	13 (27.08)	0.219	6 (15.00)	13 (25.00)	0.360	5 (13.15)	14 (25.92)	0.219	8 (21.05)	11 (20.37)	1.000
Hyperlipidemia	11 (25.0)	16 (33.3)	0.517	11 (27.5)	16 (30.8)	0.912	12 (31.6)	15 (27.8)	0.872	12 (31.6)	15 (27.8)	0.872
DMV score	5 (3, 9)	9 (7, 11)	<0.001	6 (5, 10)	8 (6, 11)	0.169	6 (4, 9)	8 (6, 11)	0.020	6 (4, 9)	8 (6, 11)	0.020

*Values are expressed as frequency (percentage) or mean ± standard deviation or median (interquartile range).*

**TABLE 2:** Multivariate analysis for the presence of cerebral small vessel disease.

	**HWMH^*a*^**	***P*-value**	**CMB^*b*^**	***P*-value**	**PVS^*c*^**	***P*-value**	**LI^*d*^**	***P*-value**
Age, years	1.07 (1.01, 1.14)	0.024			1.17 (1.09, 1.27)	<0.001	0.95 (0.89, 1.01)	0.141
Grade of CMB	1.78 (1.07, 3.08)	0.017						
WMH			0.60 (0.27, 1.27)	0.197	1.50 (0.77, 3.08)	0.284	1.86 (1.00, 3.63)	0.053
Periventricular WMH					0.62 (0.14, 2.50)	0.592	0.73 (0.19, 2.76)	0.404
Deep WMH			4.26 (1.23, 17.30)	0.03	Reference		Reference	
Lacunar infarction	2.23 (0.72, 7.08)	0.085			6.21 (1.84, 24.37)	<0.001		
PVS	1.62 (0.49, 5.33)	0.383					5.26 (1.63, 18.85)	0.070
Systolic blood pressure, mmHG			1.04 (1.01, 1.07)	0.023				
Diastolic blood pressure, mmHG			1.04 (0.99, 1.10)	0.148				
DMV score	1.17 (1.01, 1.38)	0.022			0.95 (0.80, 1.13)	0.025	1.13 (0.98, 1.31)	0.017

*Values are expressed as OR (95% C.I.).*

*^*a*^Adjust for age, grade of CMB, lacunar infarction and PVS.*

*^*b*^Adjust for WMH, Deep WMH, systolic and diastolic blood pressure.*

*^*c*^Adjust for age, WMH, Deep WMH, periventricular WMH and lacunar infarction.*

*^*d*^Adjust for age, WMH, Deep WMH, periventricular WMH and PVS.*

#### Deep Medullary Veins Score and CMB

After univariate analysis, there were significant differences in WMH, deep WMH, systolic and diastolic blood pressure between patients with (*n* = 52) and without (*n* = 40) CMB (*P* < 0.05); while it did not differ in age, sex, periventricular WMH, PVS, current smoking, alcohol use, diabetes mellitus, coronary disease, hypertension, pulse pressure, hyperhomocystinemia, hyperlipidemia and DMV score between presence and absence of HWMH (*P* > 0.05). After binary logistic regression analysis, deep WMH and systolic blood pressure were independently associated with the presence of CMB (*P* < 0.05).

#### Deep Medullary Veins Score and PVS

After univariate analysis, age, WMH, periventricular WMH, deep WMH, LI, and DMV score differed significantly between patients with (*n* = 54) and without (*n* = 38) HPVS (*P* < 0.05); however, it did not differ in sex, CMB, current smoking, alcohol use, diabetes mellitus, coronary disease, hypertension, blood pressure (systolic, diastolic and pulse pressure), hyperhomocystinemia and hyperlipidemia between presence and absence of HWMH (*P* > 0.05). After binary logistic regression, age, LI, and DMV score were independently associated with HPVS.

#### Deep Medullary Veins Score and LI

After univariate analysis, there were significant differences in age, WMH, periventricular WMH, deep WMH, PVS, and DMV scores between presence (*n* = 54) and absence (*n* = 38) of LI (*P* < 0.05); while it did not differ in sex, CMB, current smoking, alcohol use, diabetes mellitus, coronary disease, hypertension, blood pressure (systolic, diastolic and pulse pressure), hyperhomocystinemia and hyperlipidemia between presence and absence of HWMH (*P* > 0.05). After binary logistic regression analysis, the DMV score was independently associated with the presence of LI (*P* < 0.05).

### Deep Medullary Veins Score and Burden of Cerebral Small Vessel Disease

Clinical and imaging characteristics and comparisons among study patients according to CSVD burden are shown in Table 3. There were significant differences in DMV scores and systolic and diastolic blood pressures according to the severity of CSVD (*P* < 0.05). After ordinal logistic regression analysis, the DMV score was independently associated with the CSVD burden [OR 95% C.I., 1.227 (1.096–1.388)] (Table 4).

**TABLE 3:** Clinical and imaging characteristics and comparisons among study patients according to the burden of cerebral small vessel disease.

	**Burden of cerebral small vessel disease**			***P*-value**
	**Mild (*n* = 33)**	**Moderate (*n* = 21)**	**Severe (*n* = 38)**	
Age, years	62 ± 12	65 ± 10	68 ± 10	0.147
Sex, M	20 (60.6)	17 (81.0)	27 (71.1)	0.276
**Risk factors for CVSD**				
Current smoking	11 (33.3)	10 (47.6)	17 (44.7)	0.498
Alcohol use	7 (21.2)	8 (38.1)	18 (47.4)	0.070
Diabetes mellitus	15 (45.5)	12 (57.1)	22 (57.9)	0.532
Coronary disease	10 (30.3)	2 (9.5)	9 (23.7)	0.205
Hypertension	27 (81.8)	17 (81.0)	34 (89.5)	0.574
**Blood pressure, mmHG**				
Systolic	144 ± 18*	144 ± 22^#^	155 ± 20*^#^	0.028
Diastolic	83 ± 11*	86 ± 12	91 ± 12*	0.025
Pulse pressure	60 ± 16	58 ± 15	64 ± 16	0.336
Hyperhomocysteinemia	4 (12.1)	5 (23.8)	10 (26.3)	0.311
Hyperlipidemia	10 (30.3)	5 (23.8)	12 (31.6)	0.812
DMV score	6 (3, 9)*	7.00 (5, 9)^#^	9 (6, 12)*^#^	0.002

Values are expressed as frequency (percentage) or mean ± standard deviation or median (interquartile range).

* and ^#^ indicated comparison between the two groups and P-value < 0.05.

**TABLE 4:** Multivariate analysis for the degrees of cerebral small vessel disease.

	**OR**	**OR value (95% C.I.)**		***P*-value**
		**Lowest**	**Highest**	
DMV score	1.227	1.096	1.388	<0.001
Systolic blood pressure, mmHG	1.011	0.987	1.036	0.381
Diastolic blood pressure, mmHG	1.035	0.993	1.082	0.105

## Discussion

In this study, we found that the DMV score was associated with the presence and severity of CSVD. Decreasing visibility of the DMV yields increasing DMV scores, and the higher the DMV score, the greater the possibility of severe CSVD. Therefore, we demonstrate that the DMV is a biomarker of presence and burden of CSVD.

Although the total CSVD score summarizes the overall changes to brain tissue suggested by the neuroimaging markers and reflects the severity of CSVD simply, the total CSVD score still only considers the MRI abnormalities (Shaaban and Molad, 2020). It cannot directly reflect the pathological development of CSVD at the level of small blood vessels. At present, researchers generally believe that the pathological changes in small blood vessels appear before the MRI-based neuroimaging markers, and timely detection of these changes can facilitate early diagnosis of CSVD and merit prompt corresponding treatment.

SWI is an MRI sequence that is particularly sensitive to paramagnetic compounds such as deoxyhemoglobin. Low oxygen saturation and high levels of deoxyhemoglobin are characteristic of venous blood in the human body. Therefore, SWI allows visualization of veins without any contrast agent, and the signal-to-noise ratio is high. The DMV is the main vein responsible for draining the tissue fluid from the white matter area around the bilateral ventricles. Most DMVs run horizontally, and there are few developmental variations, so they are very suitable for researchers to observe and evaluate. The DMV score is a new option for semi-quantitative assessment of the DMV (Zhang et al., 2017). In this study, the agreement between readers for the DMV score was excellent. Moreover, we found that the DMV score was associated with the presence and severity of CSVD. The main possible explanations follow.

From a pathological point of view, the decreased visibility of DMV in SWI may reflect periventricular venous collagenosis (PVC). PVC is a neurodegenerative disease involving remodeling of vein wall related to aging and atherosclerosis (Moody et al., 1995; Keith et al., 2017). PVC manifests as the concentric deposition of type I and type III collagen in the walls of the veins around the ventricles, resulting in venous wall thickening, stenosis, and occlusion (Keith et al., 2017), under which the DMV signal would demonstrate discontinuous or decreased visibility on SWI. Other authors also believe that PVC plays an important role in the pathogenesis of CSVD, and PVC is closely related to the pathological changes in HWMH and periventricular LI (Keith et al., 2017). This further supported our hypothesis that the DMV score reflects the pathological process of CSVD and is correlated with the severity of CSVD.

From the perspective of hemodynamics, a high DMV score may be associated with decreased cerebral blood flow (CBF) in patients with CSVD (Shi et al., 2016; Shaaban and Molad, 2020). Decreased CBF is related to the severity of CSVD, and is one of the main findings in CSVD (Shi et al., 2016; Fulop et al., 2019). The brain needs a continuous and non-pulsating blood supply to maintain its normal physiological environment. Both the cerebrospinal fluid and the intracerebral venous system play a critical role in dampening arterial blood flow pulsation (Fulop et al., 2019). However, with aging and appearance of hypertension, the pulsation of arterial blood flow increases, and the high-pulsation blood flow is transported to the brain vessels, increasing the burden of the veins. Thus, the venous pressure increased. Sustained venous hypertension would cause retrograde venous blood flow and changes, such as PVC, in the venous wall (Rivera-Rivera et al., 2017). Resistance to blood flow is further increased, which promotes further reduction of CBF, aggravates ischemia in the brain tissue, and finally produces a vicious cycle of insufficient blood supply in the brain. Chronic hypoperfusion and hypometabolism in CSVD contribute to inadequate blood supply in a narrowing DMV and to relatively low oxygen extraction, which presents as decreased visibility of the DMV.

In integrating the above pathological and hemodynamic changes of CSVD, we believe the DMV plays a key role in the formation and progression of CSVD, and its mechanism needs further study.

Moreover, with respect of CSVD, DMV also attracted some other researchers’ attention. Shaaban et al. (2017) found DMV changes related to CSVD at 7T MRI initially. Subsequently, it was also detected at 3T MRI (Yan et al., 2014; Zhang et al., 2017; Zhou et al., 2020). The assessment method of DMV was different, such as DMV scoring system, DMV volume. Nevertheless, their results were consistent with our study partly. However, most previous reports just analyzed the relationship of DMV to a single neurobiomarker of CSVD. CSVD is a kind of clinical system, CSVD burden or total CSVD score is better to reflect the process of CSVD. Although Chen et al. (2020) demonstrated DMV score was associated with CSVD burden, it mainly highlighted the risk factors of visibility of DMV. We not only explored its correlation with each imaging biomarker of CSVD according to STRIVE, but also summarized its role in total score or burden of CSVD, highlighting the presence and burden of CSVD. Moreover, Yan et al. (2014) found that the volume of DMV is higher in patients with WMH than controls, which is inconsistent with our study. In this study, it indicated that higher burden of CSVD, more decreased visibility of DMV. Thus, we thought that the changes of DMV related to CSVD is a gradual progression. At earlier stage, DMVs were present prominently due to increased venous pressure and compensatory dilatation. With progression of CSVD, DMV changed from compensatory dilatation to collagen deposition, lumen stenosis, and even complete occlusion.

Our study has several limitations. First, we enrolled only a small number of patients in a single center. Larger numbers of patients from multiple centers should be evaluated in the future. Second, there is no brain perfusion imaging, which would be helpful to interpret the role of the DMV score in CSVD. Third, the DMVs were measured with direct visualization according to a qualitative scoring system. There are a number of reasons why a vein may be less visible or why the signal may appear to be non-continuous. Poor visibility/lack of continuity could also simply be due to the MRI slice angle. Thus, a quantitative method, such as quantitative susceptibility mapping, may be better to evaluate DMV. Forth, we did not measure any changes in blood vessel cell markers and signaling peptides. Finally, we did not finish a long-term follow- up for cognitive decline and dementia, which will also need further study in the future.

## Conclusion

The DMV score was associated with the presence and burden of CSVD. Thus, we hypothesize that DMV is an easy-useful biomarker of presence and burden of CSVD.

## Data Availability Statement

The original contributions presented in the study are included in the article/supplementary material, further inquiries can be directed to the corresponding author/s.

## Ethics Statement

The studies involving human participants were reviewed and approved by the General Hospital of Northern Theater Command. The patients/participants provided their written informed consent to participate in this study.

## Author Contributions

ZX, FL, and YD conceived the project idea and wrote the manuscript. BY provided critical suggestions for the experiments design. FL, BW, DX, and YP collected the imaging and clinical data. ZX, FL, BW, and BY provided the imaging analysis. YD and BY supervised the project. All authors contributed to the article and approved the submitted version.

## Conflict of Interest

The authors declare that the research was conducted in the absence of any commercial or financial relationships that could be construed as a potential conflict of interest.
